# Abnormal dynamic resting-state brain network organization in auditory verbal hallucination

**DOI:** 10.1007/s00429-020-02119-1

**Published:** 2020-08-19

**Authors:** Haiyang Geng, Pengfei Xu, Iris E. Sommer, Yue-Jia Luo, André Aleman, Branislava Ćurčić-Blake

**Affiliations:** 1 Center for Brain Disorders and Cognitive Sciences, Shenzhen University, Shenzhen, China; 2 Department of Biomedical Sciences of Cells and Systems, University of Groningen, University Medical Center Groningen, Groningen, The Netherlands; 3 Beijing Key Laboratory of Applied Experimental Psychology, Faculty of Psychology, Beijing Normal University, Beijing, China; 4 Center for Emotion and Brain, Shenzhen Institute of Neuroscience, Shenzhen, China; 5 Great Bay Neuroscience and Technology Research Institute (Hong Kong), Kwun Tong, Hong Kong China; 6 Sichuan Center of Applied Psychology, Chengdu Medical College, Chengdu, China

**Keywords:** Auditory-verbal hallucinations, Dynamic connectivity, Network-antagonistic state, Default model network, Language network

## Abstract

Auditory-verbal hallucinations (AVH) are a key symptom of schizophrenia. Recent neuroimaging studies examining dynamic functional connectivity suggest that disrupted dynamic interactions between brain networks characterize complex symptoms in mental illness including schizophrenia. Studying dynamic connectivity may be especially relevant for hallucinations, given their fluctuating phenomenology. Indeed, it remains unknown whether AVH in schizophrenia are directly related to altered dynamic connectivity within and between key brain networks involved in auditory perception and language, emotion processing, and top-down control. In this study, we used dynamic connectivity approaches including sliding window and *k*-means to examine dynamic interactions among brain networks in schizophrenia patients with and without a recent history of AVH. Dynamic brain network analysis revealed that patients with AVH spent less time in a ‘network-antagonistic’ brain state where the default mode network (DMN) and the language network were anti-correlated, and had lower probability to switch into this brain state. Moreover, patients with AVH showed a lower connectivity within the language network and the auditory network, and lower connectivity was observed between the executive control and the language networks in certain dynamic states. Our study provides the first neuroimaging evidence of altered dynamic brain networks for understanding neural mechanisms of AVH in schizophrenia. The findings may inform and further strengthen cognitive models of AVH that aid the development of new coping strategies for patients.

**Electronic supplementary material:**

The online version of this article (10.1007/s00429-020-02119-1) contains supplementary material, which is available to authorized users.

## Introduction

Auditory-verbal hallucinations (AVH) are perceptual experiences in the absence of external auditory-verbal stimuli, which are sufficiently compelling to be considered as true perceptions. AVH are a characteristic symptom in schizophrenia, and approximate 70% of patients report AVH at some point in their illness course (Slade and Bentall 1988). A particular characteristic of AVH is that they show dynamic fluctuations in their occurrence (Nayani and David 1996; McCarthy-Jones et al. 2014). Despite decades of investigations, neural mechanisms underlying hallucinations remain unclear (Ćurčić-Blake et al. 2017a). In recent years, resting-state functional connectivity has been commonly used in functional Magnetic Resonance Imaging (fMRI) studies to characterize interactions between brain networks in schizophrenia patients. These studies reported evidence of extensive disruption of brain networks including cortico-thalamo, fronto-limbic, and fronto-temporal connectivity in patients (Sarpal et al. 2015; Sheffield and Barch 2016; van den Heuvel et al. 2016; Chang et al. 2017), consistent with the hypothesis of schizophrenia as a disorder of brain disconnection (Stephan et al. 2009). Currently, there have been over 60 studies illustrating alterations in functional and anatomical connectivity in schizophrenia patients with AVH (Ćurčić-Blake et al. 2017a), which are involved in auditory perception, language, emotion, memory, and top–down control (Rotarska-Jagiela et al. 2010; Jardri et al. 2011; Zmigrod et al. 2016; Zhang et al. 2018). While there are studies looking into dynamic changes in interactions within and between brain networks in schizophrenia patients (Damaraju et al. 2014; Du et al. 2016; Su et al. 2016), it is very relevant and significant to investigate such dynamic changes specifically in AVH which have a fluctuating nature.

Our previous model of hallucinations (Aleman et al. 2003; Allen et al. 2008b) proposed that abnormal interaction between top–down and bottom–up processes may underlie AVH (Behrendt 1998; Grossberg 2000; Corlett et al. 2019). In particular, we suggested that spontaneous hyperactivity of the superior temporal gyrus may contribute to ‘over-perceptualization’, accompanied by reduced modulation from Broca's areas involved in language processing and the anterior cingulate cortex (ACC), the Supplementary Motor Area (SMA) involved in monitoring speech (Allen et al. 2008b). Over the past decades, a number of resting-state and task fMRI studies have shown altered function of language, auditory networks and emotion, executive control, and default mode networks in AVH of schizophrenia patients (see Ćurčić-Blake et al. 2017, for a review). Some of these studies found altered fronto-temporal connectivity in the language network across different language tasks (Lawrie et al. 2002; Hashimoto et al. 2010) as well as during resting state (Mechelli et al. 2007) in schizophrenia patients with AVH. Additionally, aberrant connectivity in the default mode network (DMN) and the auditory network have been shown to be related to severity of hallucinations (Rotarska-Jagiela et al. 2010), which may be linked to self-related processing, intrusion of language-related memory and auditory processing, respectively (Northoff and Qin 2011). Moreover, some studies showed that AVH in schizophrenia were related to altered activation and connectivity of the emotion network including the amygdala and cingulate cortex during processes of auditory and emotional stimuli (Kang et al. 2009; Escartí et al. 2010; Amad et al. 2014). Finally, besides roles of the auditory and language networks in production of auditory verbal hallucinations, the executive control network, including the prefrontal and parietal cortex, is thought to be essential for inhibitory control and attention to the voice in AVH (Hugdahl 2009; Alderson-Day et al. 2015). It currently remains unknown to which extent dynamic (i.e., time-varied) interactions within and between these key networks contribute to AVH.

The human brain shows a highly dynamic functional activity and connectivity. This dynamic nature of brain provides the backbone for a variety of complex and flexible cognitive processes (Hutchison et al. 2013; Shine et al. 2016), which have been found abnormal in mental disorders (Damaraju et al. 2014; Kaiser et al. 2016). Typical static resting-state functional connectivity reflects the correlation between averaged time courses of brain regions, which has an implicit but oversimplified assumption of spatial and temporal stationarity throughout entire scanning period (Hutchison et al. 2013; Allen et al. 2014). In contrast, dynamic connectivity analysis takes temporal fluctuations within one scanning session into account by calculating the variability of functional connectivity over time. Dynamic functional connectivity has several merits over static connectivity for investigating neural mechanisms underlying symptoms of mental disorders including hallucinations. First, dynamic functional connectivity measures have been found more informative about various aspects of brain connectivity, which outperformed static connectivity for classifying schizophrenia and healthy controls (Rashid et al. 2016). More importantly, dynamic connectivity analysis, especially clustering approaches such as sliding window and k-means, can look into high-order statistics of brain dynamics, dwelling, and switching within and between dynamic brain states (or cognitive states). It can resolve different brain connectivity patterns corresponding to distinct mental processes (Gonzalez-Castillo et al. 2015), which may be useful for examining fluctuation of hallucinations (Nayani and David 1996; Kindler et al. 2011; Lefebvre et al. 2016), and that cannot be detected by static connectivity analyses. Finally, it has been shown that the differences in connectivity between patients and controls may not be localized in a single dynamic state, but distributed across different dynamic states. The static functional connectivity calculates the averaged connectivity for the entire scanning session and might not detect distributive nature of the group differences (Rashid et al. 2014).

Indeed, dynamic connectivity analyses have provided new evidence to understanding brain dynamics in schizophrenia, but no fMRI studies have investigated dynamic connectivity in AVH patients. These studies in schizophrenia have indicated that patients showed alteration in the dynamic graph metrics as well as functional connectivity primarily in fronto-parietal and temporal lobe regions compared to healthy controls (HCs) (Ma et al. 2014; Yu et al. 2015). For dwelling and switching among dynamic brain states, schizophrenia patients were found to spend less time in an integrated state and more time in a weak connectivity state (Damaraju et al. 2014; Du et al. 2016), consistent with disconnection hypothesis of schizophrenia. Importantly, abnormal functional connectivity patterns are more pronounced during these dynamic brain states showing altered dwell times (Damaraju et al. 2014). Additionally, schizophrenia patients showed alterations in connectivity during one specific dynamic brain state (out of 5 states), where the DMN regions showed mostly asynchronous activation with other functional networks (Rashid et al. 2014). Phenomenological surveys and EEG studies have found that AVH are also very dynamic on different time scales across sub-minutes, days, and weeks (Nayani and David 1996; Koenig et al. 1999; Kindler et al. 2011; McCarthy-Jones et al. 2014). EEG studies showed that in a specific microstate with a fronto-central distribution was shorter in schizophrenia patients when compared to HCs (Koenig et al. 1999; Kindler et al. 2011). Notably, this shortening was correlated to positive psychotic symptoms including AVH (Kindler et al. 2011). However, considering limited space resolution of EEG, the dynamic interactions between key brain networks associated with AVH remain unknown. In one fMRI study, Lefebvre et al. used ‘dynamic causal model analysis’ (DCM) to examine the causal interaction between brain networks during distinct periods of the emergence of hallucinations. The hallucination periods were inferred using independent component analysis (ICA) (Lefebvre et al. 2016). They found involvement of the salience network, relevant for switching between inner thought and external world. In contrast, data-driven approaches such as clustering (e.g., k-mean) can be used to infer the neural or cognitive state based on functional connectivity patterns. It might provide a more objective way to characterize the dynamic interactions between key brain networks which are involved in AVH. Such networks include the language network, the auditory network, the default-mode network, the emotion network, and the executive control network, which play key roles in AVH (Allen et al. 2008b; Ćurčić-Blake et al. 2017a).

In the present study, we used dynamic connectivity analysis including sliding window and k-means to unveil the dynamic characterization of interactions among key brain networks related to AVH in schizophrenia patients. Static connectivity analysis was also used to complement and be compared with results from dynamic connectivity analysis. We expected that inter- and intra-network connectivity would exhibit alterations in connectivity patterns during certain dynamic brain states in AVH patients, especially within the auditory and language networks as well as between these two networks and other networks. Differences in network connectivity between the two groups might be most pronounced in the brain states which showed differences in dwell times and switching. These hypothesized alteration of brain dynamic in AVH patients may be of relevance to fluctuations that characterize hallucinatory activity.

## Materials and methods

### Participants

Schizophrenia patients with (*n* = 22) and without (*n* = 17) a recent history of AVH were included in the present study. Only right-handed patients were recruited, because handedness has previously been proven to influence the brain lateralization (Parker et al. 2005). These patients are selected from three datasets at our institute (Pijnenborg et al. 2011; Liemburg et al. 2015; Lange et al. 2015) if there was a clear information about current history of AVH. They were further divided into the AVH and non-AVH groups according to item M6 (Have you ever heard things other people couldn't hear?) of the Mini International Neuropsychiatric Interview-Plus (MINI) and the patients’ dossiers (Sheehan et al. 1998). For the 20 patients, MINI interview data were available. If the MINI was not available, we consulted the principal clinician and the patient dossier. Only if it was possible to confirm with certainty that participants did not experience AVH in their life (12 out of 17 patients) or not recently (i.e., in the last 6 months, 5 of 17 patients), they were included in the non-AVH group. In contrast, if it was certain that they did experience AVH in the last 6 months (22 patients, 15 of which experienced AVH in the week before fMRI scanning), we included them in the AVH group. Noteworthy, there were five patients in the non-AVH group who experienced hallucinations in other modalities (tactile, olfactory, or presence), but never had AVH. This is why some patients in the non-AVH group had P3 >  = 3. The severity of symptoms was also assessed using the Positive and Negative Syndrome Scale (PANSS) interview (Kay et al. 1987). Medication effects were examined according to a standardized quantitative method for comparing dosages of different drugs (Andreasen et al. 2010). Each medication dose was expressed in equivalent doses of haloperidol. The study was approved by the medical ethics committee of the University Medical Center Groningen and performed according to the Declaration of Helsinki.

### Imaging data acquirement and preprocessing

Magnetic resonance images were acquired by a 3 T Phillips Intera Quaser MRI scanner (Philips Intera, Best, The Netherlands). The fMRI data came from three datasets (Pijnenborg et al. 2011; Liemburg et al. 2015; Lange et al. 2015). All functional images were collected with single-shot gradient-recalled echo planar imaging (GR-EPI) sequences, aligned along the anterior commissure-posterior commissure (AC-PC) line. Nine AVH patients and eight non-AVH patients came from the dataset one (200 scans; TR = 1500 ms, TE = 28 ms, FA = 85°, matrix = 64 × 62, FOV = 220 mm, 3 mm slice thickness, 0 mm spacing between slices, 43 transverse slices), none AVH patient and five non-AVH patients from the dataset two (200 scans; TR = 2300 ms, TE = 28 ms, FA = 85°, matrix = 64 × 62, FOV = 220 mm, 3 mm slice thickness, 0 mm spacing between slices, 43 transverse slices), and thirteen AVH patients and four non-AVH patients form the dataset three (200 scans; TR = 2000 ms, TE = 30 ms, FA = 70°, matrix = 64 × 62, FOV = 220 mm, 3 mm slice thickness, 0 mm spacing between slices, 37 transverse slices). For spatial normalization, T1-weighted anatomical images were collected in axial orientation using a 3D gradient-recalled sequence (251 scans; TR: 9 ms; TE = 3.5 ms, FA = 8°, slice thickness = 1 mm, FOV = 232 mm, matrix = 256 × 256, 170 transverse slices) on each subject.

Dpabi software was used to perform resting-state preprocessing and connectivity analysis (Yan et al. 2016). Slice timing correction, realignment and spatial normalization and smoothing (full-width-half-maximum, FWHM = 6 mm) were conducted. Considering that the head motion could impact connectivity, we excluded two patients who have head motion more than 3 mm and 3 degrees. The 22 patients with AVH and 17 patients without AVH were included in the final analysis. Then, we calculated frame-wise displacement (FD) from the derivatives of the six rigid-body realignment parameters estimated during standard volume realignment, as well as the root-mean-square change in BOLD signal from volume to volume (DVARS). The outliers were replaced by missing values corrected using linear interpolation. This approach has been widely used in resting-state fMRI study (Power et al. 2017), especially in dynamic connectivity analysis (Shine et al. 2016) to assure the continuity of the brain dynamics. After artifact detection, we regressed out nuisance covariates including 12 linear head movement parameters (and their temporal derivatives), FD and DVARS, as well as the noises from the CSF and WM using the CompCor strategy (Behzadi et al. 2007). Finally, data were de-trended and filtered (0.01–0.08 Hz).

### Key nodes in three networks as regions of interest

**Fig. 1:**
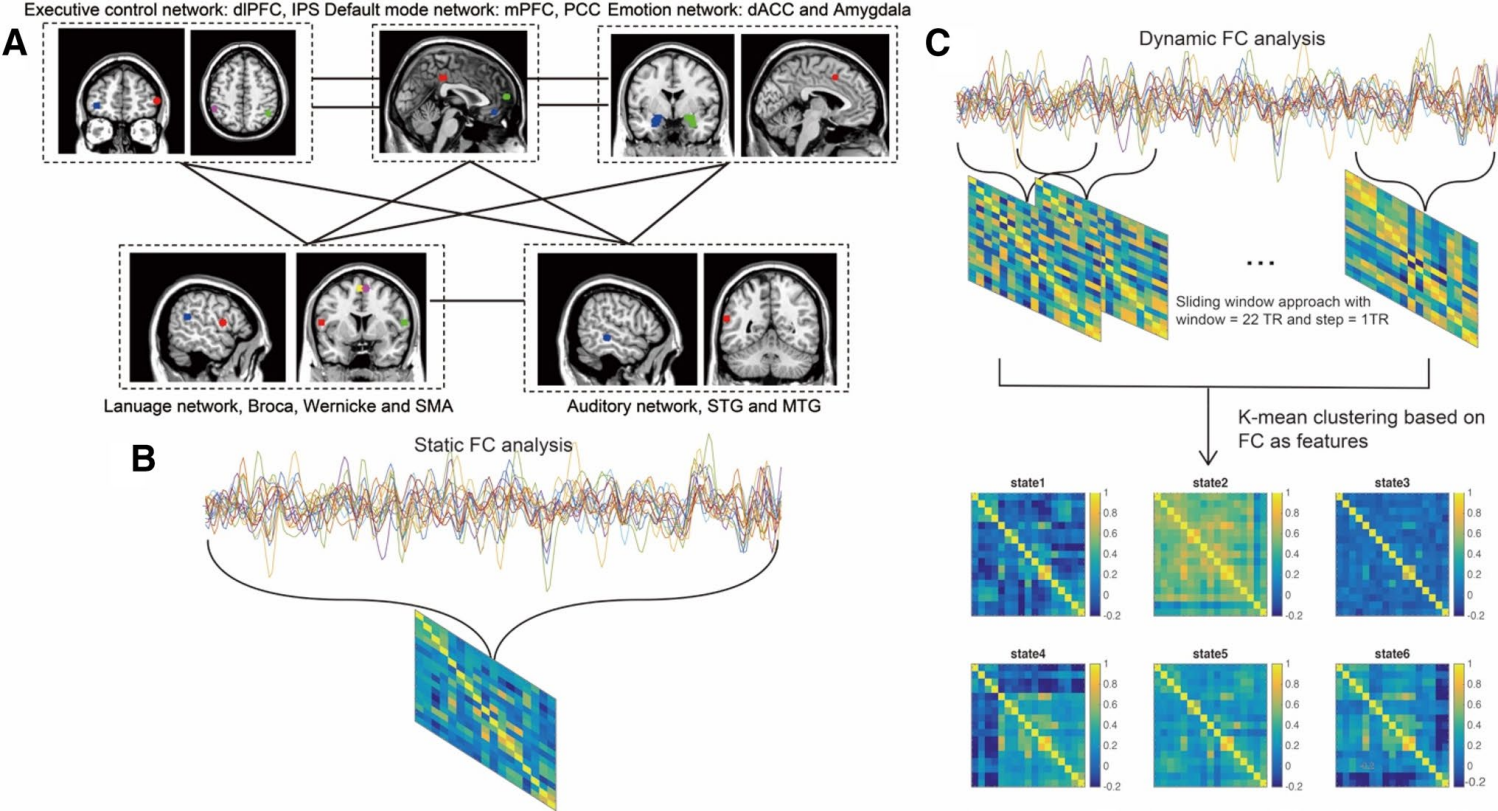
The pipeline of static and dynamic connectivity analysis. **a** An illustration of inter- and intra-network interaction using predefined regions of interest involved in auditory-verbal hallunicition. **b** Static and **c** dynamic functional connectivity analysis. Notes: *dlPFC* dorso-lateral prefrontal cortex, *IPS* inferior parietal sulcus, *mPFC* medial prefrontal cortex, *PCC* posterior cingulate cortex, *dACC* dorsal anterior cingulate cortex, *STG* superior temporal gyrus, *MTG* middle temporal gyrus, *FC* functional connectivity

The regions of interest (ROIs) were produced using coordinates in previous influential meta-analysis (Jardri et al. 2011; Zmigrod et al. 2016) and experimental fMRI studies (Qin et al. 2012; McMenamin et al. 2014) (Fig. 1a; Table 1). Every region was defined as a 6 mm radius sphere. Left Broca’s region and left Wernicke’s region, right IFG were chosen as the nodes in the language network. Right middle temporal gyrus and left superior temporal gyrus were chosen as nodes in the auditory network (Jardri et al. 2011; Zmigrod et al. 2016). Left and right medial prefrontal cortex (MPFC), medial posterior cingulate cortex (PCC) in the default mode network (DMN), left and right dorsal-lateral prefrontal cortex (dlPFC) and left and right inferior parietal sulcus (IPS) in the executive control network (ECN), and medial dorsal cingulate cortex (dACC) (McMenamin et al. 2014) and left and right amygdala (Qin et al. 2012) were chosen as nodes in the emotion network.

**Table 1:** Coordinates of ROIs

Regions	*x*	*y*	*z*	Networks
dlPFC L	– 35	49	6	ECN
dlPFC R	47	46	13	ECN
IPS L	– 48	− 51	52	ECN
IPS R	37	− 60	48	ECN
PCC	– 3	− 39	39	DMN
MFG L	– 3	44	− 2	DMN
MFG R	2	57	24	DMN
dACC	0	12	46	Emotion
Amygdala L	– 24	0	– 18	Emotion
Amygdala R	24	0	– 18	Emotion
Broca area L	– 56	4	12	Language
Wernicke area L	– 58	– 46	20	Language
IFG R	60	8	12	Language
SMA L	– 2	8	60	Language
SMA R	6	6	60	Language
STG L	– 60	– 56	20	Auditory
MTG R	60	– 32	– 6	Auditory

All the coordinates are in MNI atlas space. Notes *dlPFC* dorso-lateral prefrontal cortex, *IPS* inferior parietal sulcus, *mPFC* medial prefrontal cortex, *PCC* posterior cingulate cortex, *dACC* dorsal anterior cingulate cortex, *STG* superior temporal gyrus, *MTG* middle temporal gyrus, *ECN* executive control network, *DMN* default mode network

### Dynamic functional connectivity analysis

Dynamic functional connectivity between key nodes was computed using a sliding window approach with a window size of 22 TRs in steps of 1 TR (Allen et al. 2014), functional connectivity (Pearson correlation) between these ROIs was calculated one by one in each window (Fig. 1c). Then, we used a *k*-means algorithm to cluster these dynamic FC windows with functional connectivity as features, by partitioning the data into a set of separate clusters so as to maximize the correlation within a cluster to the cluster centroid. We determinated number of clusters as six by using Dunn index ( $DI_{m} = \mathop {\min }\nolimits_{{0 \le i < j \le m}} \delta \left( {C_{i} ,c_{j} } \right)/\mathop {\max }\nolimits_{{1 \le k \le m}} \vartriangle _{k}$ ) (see the Supplemental Materials). After dividing all the time windows into six distinct FC states, in each state, the intra- and inter-network FC were calculated by averaging FC within and between networks. To quantify the within-network connectivity, we averaged all the connectivity values between the regions within a network and used the mean as a representative of within-network connectivity. Similar approach was used to calculate the mean of connectivity between two different brain networks to represent between-network connectivity. Noteworthy, before averaging, we checked the FC distributions within and between networks, and they are approximately normally distributed, and thus, it was reasonable to calculate the mean values. For follow-up analysis, we selected State 3 and State 6, because they showed different dwell times between the two groups. To characterize the meaning of brain states (i.e., State 3 and State 6), weighed connectivity matrix was used to calculate graph theory measurements including shortest path length and clustering coefficient (Wang et al. 2015), which was further compared across states. We also compared functional connectivity between the default mode network and other networks, especially the language network across states. For the dwell times, we used npIntFactRep (R package) to do a nonparametric aligned rank test for examining interaction in two-way factorial design with Group as the between subject factor (AVH vs. non-AVH group) and State as the within-subject factor (State 3 vs. State 6). Furthermore, we compared the dwell times of State 3 and State 6 between patients with AVH and without AVH using nonparametric permutation test, and we calculated and transformed the transition probability between different states and connectivity within and between networks (in State 3 and 6) into z scores and compared them between the two groups using two-sample *t* test. We performed *t* tests between male patients with and without AVH to investigate whether the main results hold for males.

### Static functional connectivity analysis

We also characterized the static functional connectivity (FC) of intra- and inter-network and compared differences of these FC between patients with AVH and without AVH (Fig. 1b). For each individual, the Pearson correlations of averaged time series between ROIs were calculated and transformed to *z* values. Then, the intra- and inter-network FC were computed by averaging FC within networks and between these networks, and compared between patients with and without AVH using two-sample *t* test. There are too few females in the AVH and non-AVH groups to directly examine the gender effects on the static FC during the group comparisons. However, we performed *t* tests between male patients with and without AVH to investigate whether the main results hold for males.

## Results

### Patients

AVH (Mean (SD) of age: 32.2 (11.9), gender: 14 Male/8 Female) and non-AVH (Mean (SD) of age: 32.1 (7.5), gender: 16 Male/ 1 Female)) patients did not differ in age, education, duration of illness, and haloperidol equivalents. However, two groups showed significant difference in P3 and gender (details are shown in the Table 2).

**Table 2:** Demographic data of patients

	Mean (SD)		Significance (*p* value)
	AVH (*n* = 22)	non-AVH (*n* = 17)	AVH vs non-AVH
Age	32.2 (11.9)	32.1 (7.5)	*T*(37) = 0.019 (0.985)
Male (female)	14 (8)	16 (1)	χ2(1, 37) = 5.019 (0.025)
Education	5.0 (1.0)	5.4 (1.0)	*T*(37) = – 1.415 (0.166)
P3	3.2 (1.5)	2.1 (1.5)	*T*(37) = 1.11 (0.024)
PANSS pos	14.2 (4.1)	13.5 (5.4)	*P* = 0.711 (0.626)
PANSS neg	14.3 (4.9)	15.1 (4.8)	*P* = – 0.845 (0.569)
PANSS gen	28.8 (5.8)	29.7 (9.8)	*P* = – 0.888 (0.714)
PANSS total	57.3 (11.0)	58.3 (18.5)	*P* = – 1.021 (0.826)
Duration of illness (year)	7.3 (7.6)	8.6 (8.3)	*T*(37) = – 0.515 (0.61)
Medication (mg) Haloperidol equivalent	7.1 (4.7)	5.9 (3.9)	*P* = 1.243 (0.376)

The left column lists the demographic variables. The 2nd–4th columns show average values of the variables across the group, with their standard deviations in brackets. Education level was rated according to a six-point scale defined by Verhage, which ranges from primary school (1) to university level (6). Nonparametric tests were used to test the group difference for PANSS (permutation test) and gender (Chi-square)

### Dynamic dwelling and switching

**Fig. 2:**
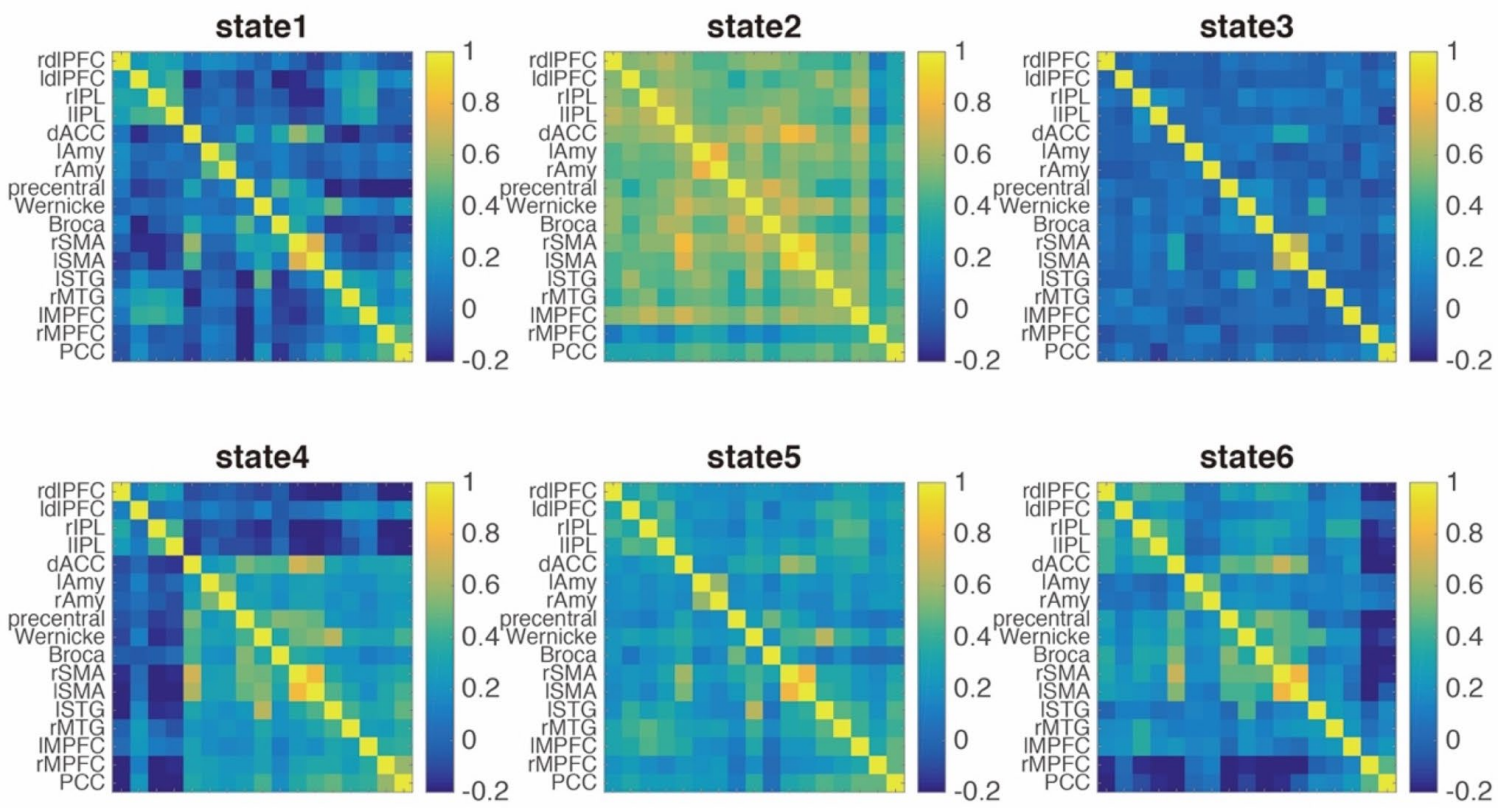
Connectivity patterns of six brain states in dynamic brain. Notes: *dlPFC* dorso-lateral prefrontal cortex, *IPS* inferior parietal sulcus, *mPFC* medial prefrontal cortex, *PCC* posterior cingulate cortex, *dACC* dorsal anterior cingulate cortex, *STG* superior temporal gyrus, *MTG* middle temporal gyrus, *FC* functional connectivity

**Fig. 3:**
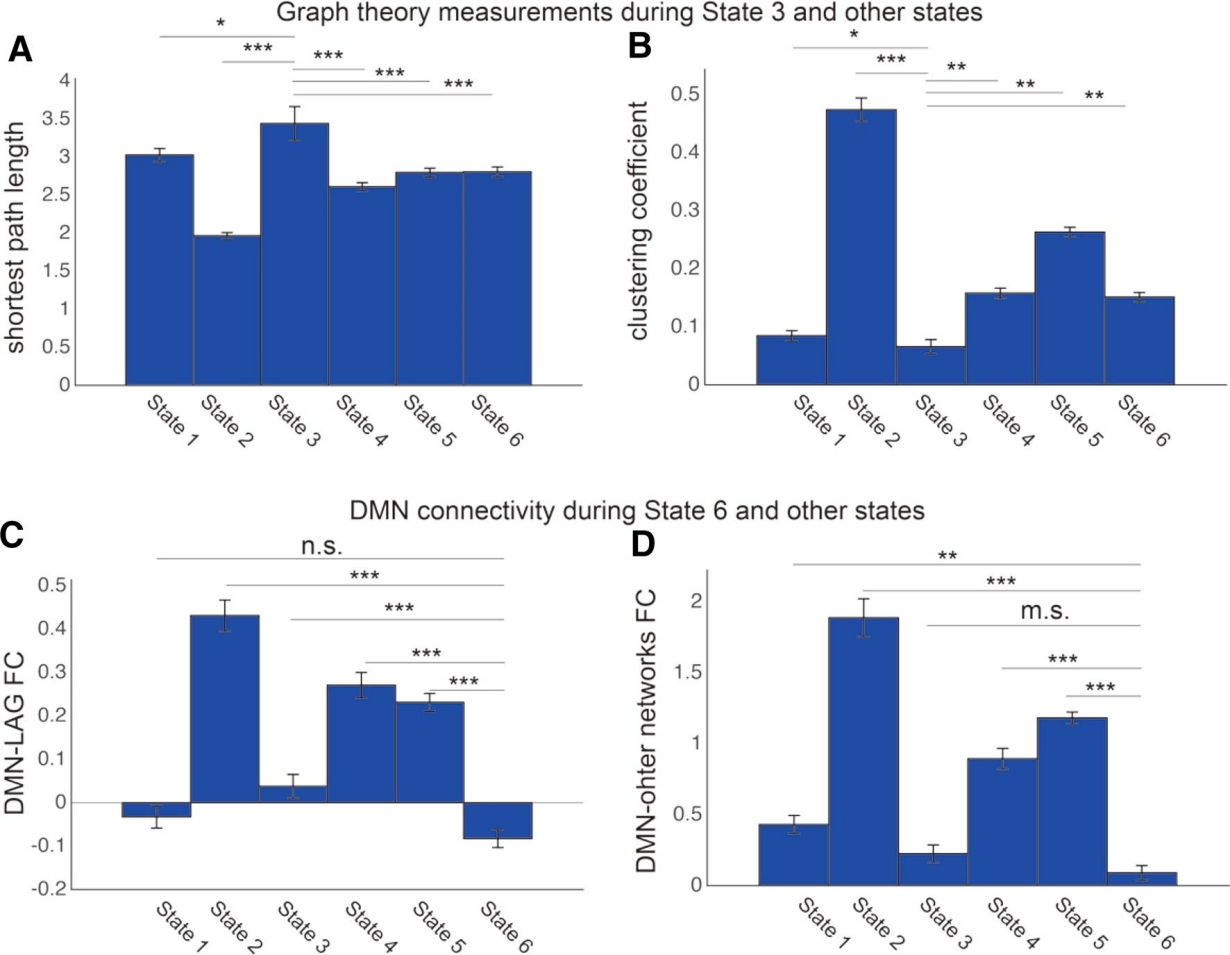
Characterizing brain states. **a–b** Graph theory measurements during State 3 compared with ones during other brain states. **c**–**d** Functional connectivity between the DMN and the language network as well as other brain network during State 6 compared with ones during other brain states. Notes: ***P < 0.001; **P < 0.01; **P < 0.05; m.s., marginally significant (*P* < 0.1); *n.s* not significant

**Fig. 4:**
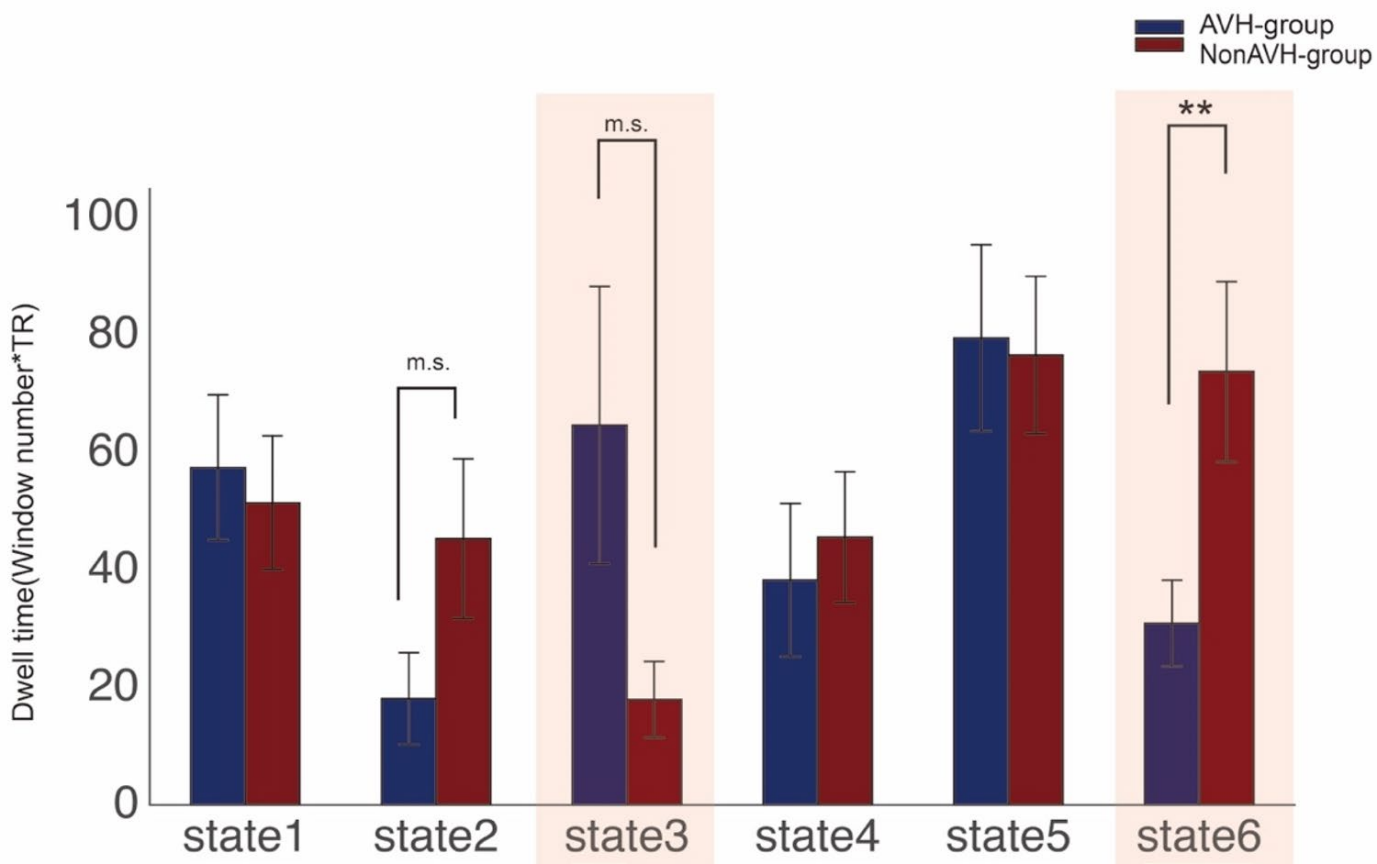
Dwell time of each brain states in AVH and non-AVH group. Notes: **P < 0.01; *m.s*., marginally significant (*P* < 0.1)

**Fig. 5:**
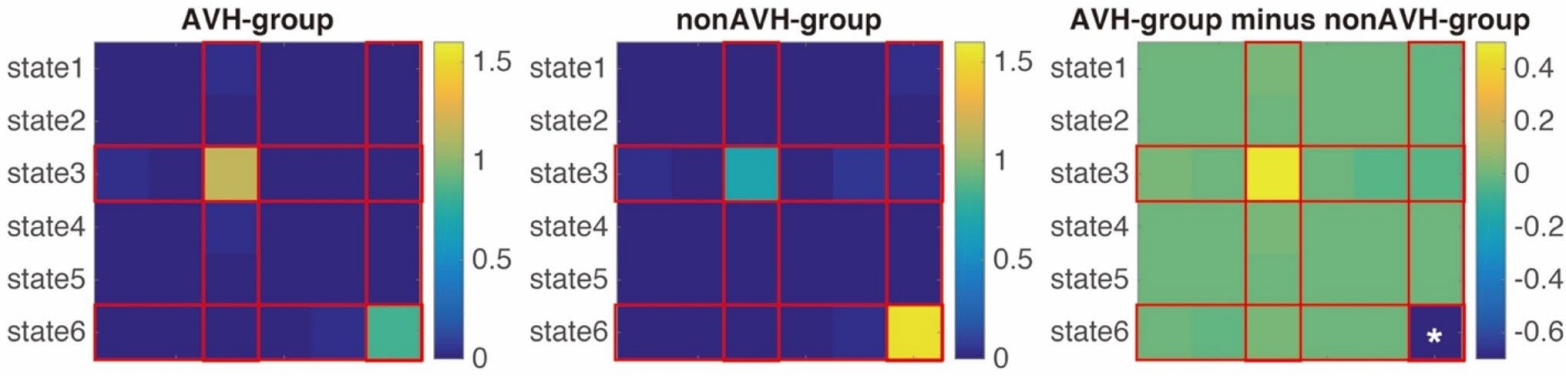
Transition probability between brain states in AVH and non-AVH groups. Notes: *P < 0.05

To investigate dynamic connectivity, we employed the sliding window and *k*-means approach to characterize dynamic interaction among key brain networks. The entire time windows were divided into six different brain states based on functional connectivity patterns. The centroid patterns are largely distinguished from each other (Fig. 2). In State 3, the brain was more segmented, which is indicated by that State 3 showed lower cluster coefficient (all *p* < 0. 038) and longer shortest path length in the graph theory analysis (all *p* < 0. 037) (See Fig. 3a–b). In the State 6, the brain showed anti-correlation between the DMN and other networks including the language network, which is suggested by that the DMN showed the strongest anti-correlation with the language network in State 6 (all *p* < 0. 001) besides compared with State 1 (*p* = 0.138) (see Fig. 3c–d). Additionally, for dwell time, interaction analysis between State (State 3 vs. 6) and Group (AVH vs. non-AVH) showed a significant interaction effect (*F*(1, 37) = 5.22, *p* = 0.03). Post hoc permutation test showed that the State 6 lasted shorter (*diff* = -42.809 windows*TR, *p* = 0.008), while State 3 appeared to last longer (*diff* = 46.707 windows*TR, *p* = 0.089) (Fig. 4) in the AVH group compared with non-AVH group. After correction for multiple comparison, the difference in dwell times between AVN and non-AVH group remained significant for the State 6 (*p*_FDR_ = 0.016). Repeated analysis in male patients showed a significant difference in State 6 (*diff* =  – 42.698 windows*TR, *p* = 0.037). Finally, we used of translation probability (TP) between brain states to measure the probability of switching between brain states. We found that AVH group showed less probability to switch from State 3 to 6 (a trend) (*t*(37) =  – 1.840, *p* = 0.074) and from 6 to itself (*t*(37) =  – 3.410, *p* = 0.002) (Fig. 5). After correction for multiple comparison, the difference of TP for the State 6 to itself between the two groups remained significant (*p*_FDR_ = 0.019). Repeated analysis in male patients showed a significant difference of TP for the State 6 to itself (*t*(28) =  – 2.859, *p* = 0.008). Spearman correlation analysis showed no significant correlation between P3 and dwell times of State 3 and State 6 as well as TP for State 3 to State 6 and State 6 to itself (all *r* < 0.204, all *p* > 0.206) in the AVH group.

### Dynamic functional connectivity

**Fig. 6:**
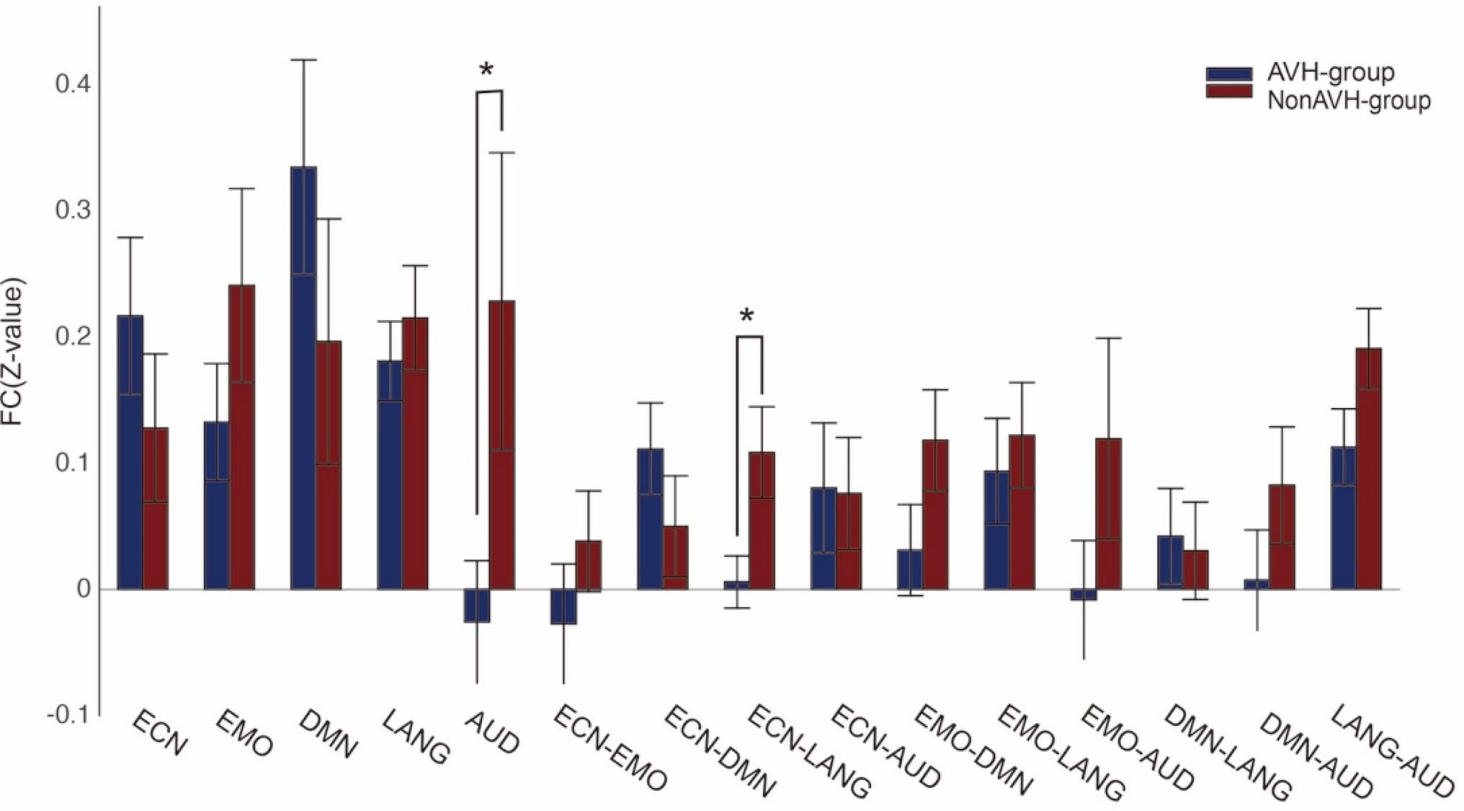
Dynamic inter- and intra-network connectivity between five core brain networks in AVH and non-AVH groups during the state 3. Notes: *ECN* executive control network, *EMO* emotion network, *DMN* default mode network, *LANG* language network, *AUD* auditory network, *P < 0.05

**Fig. 7:**
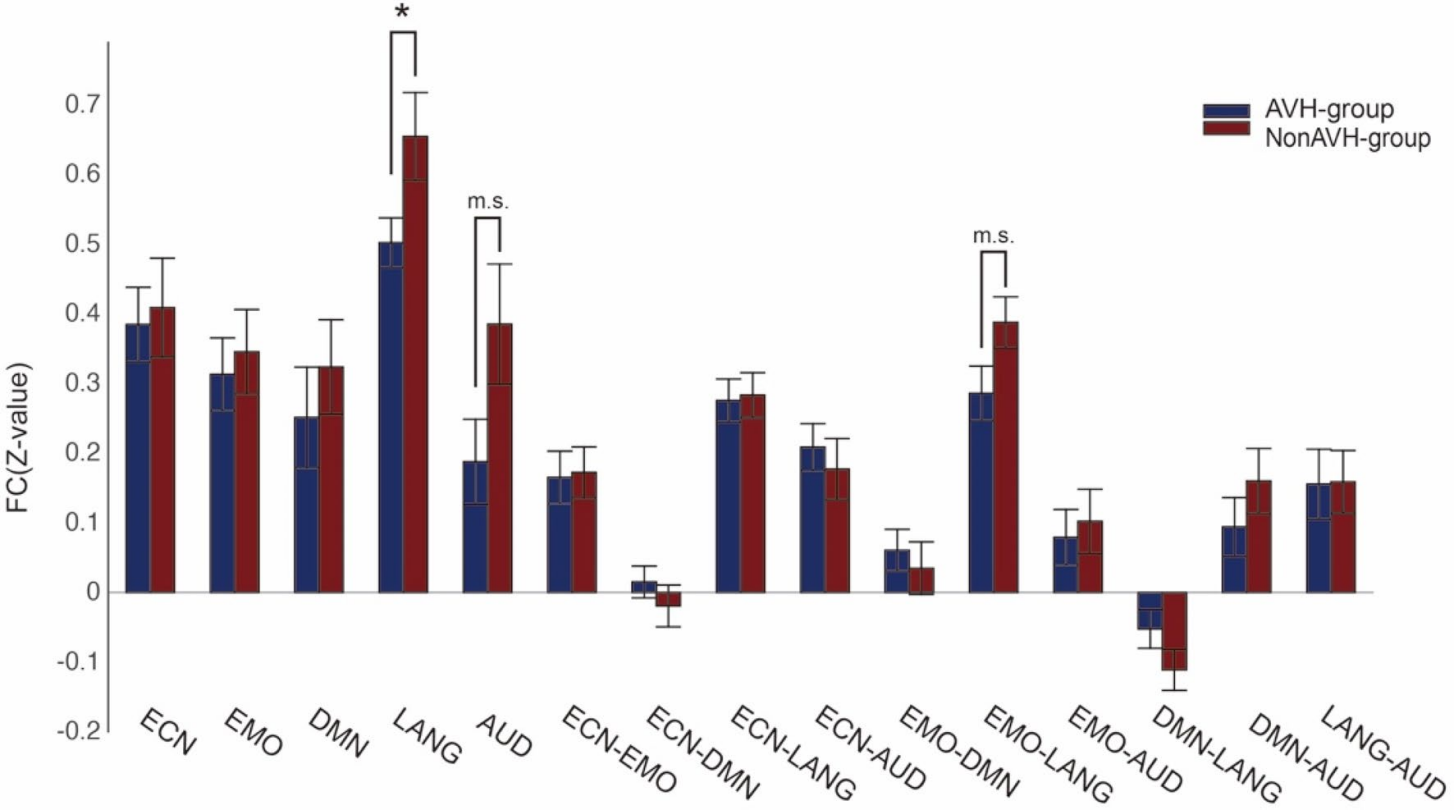
Dynamic inter- and intra-network connectivity between five core brain networks in AVH and non-AVH groups during the state 6. Notes: *ECN* executive control network, *EMO* emotion network, *DMN* default mode network, *LANG* language network, *AUD* auditory network, *P < 0.05, *m.s*., marginally significant (*P* < 0.1)

To further characterize connectivity pattern during particular dynamic brain states including State 3 and 6, we compared the inter- and intra-network connectivity during these two states between two groups. In State 3, FC within the auditory network decreased in AVH patients (*t*(37) =   – 2.320, *p* = 0.029). Repeated analysis in male patients showed a similar but marginally significant difference (*t*(28) =  – 1.757, *p* = 0.096). FC between the executive control network and the language network (*t*(37) =  – 2.669, *p* = 0.013) was decreased in AVH group (Fig. 6). Repeated analysis in male patients showed a similar significant difference (*t*(28) =  – 2.938, *p* = 0.009). In the State 6, FC was decreased in the language network in AVH group (*t*(37) =  – 2.067, *p* = 0.048) (Fig. 7). Repeated analysis in male patients showed a similar but a trend of significant difference (*t*(28) = − 1.675, *p* = 0.107). Spearman correlation analysis showed no significant correlation between P3 and connectivity within the auditory network, between the executive control network and the language network during State 3, within the language network during State 6 (all *r* < 0.219, all *p* > 0.544) in the AVH group.

### Static functional connectivity

**Fig. 8:**
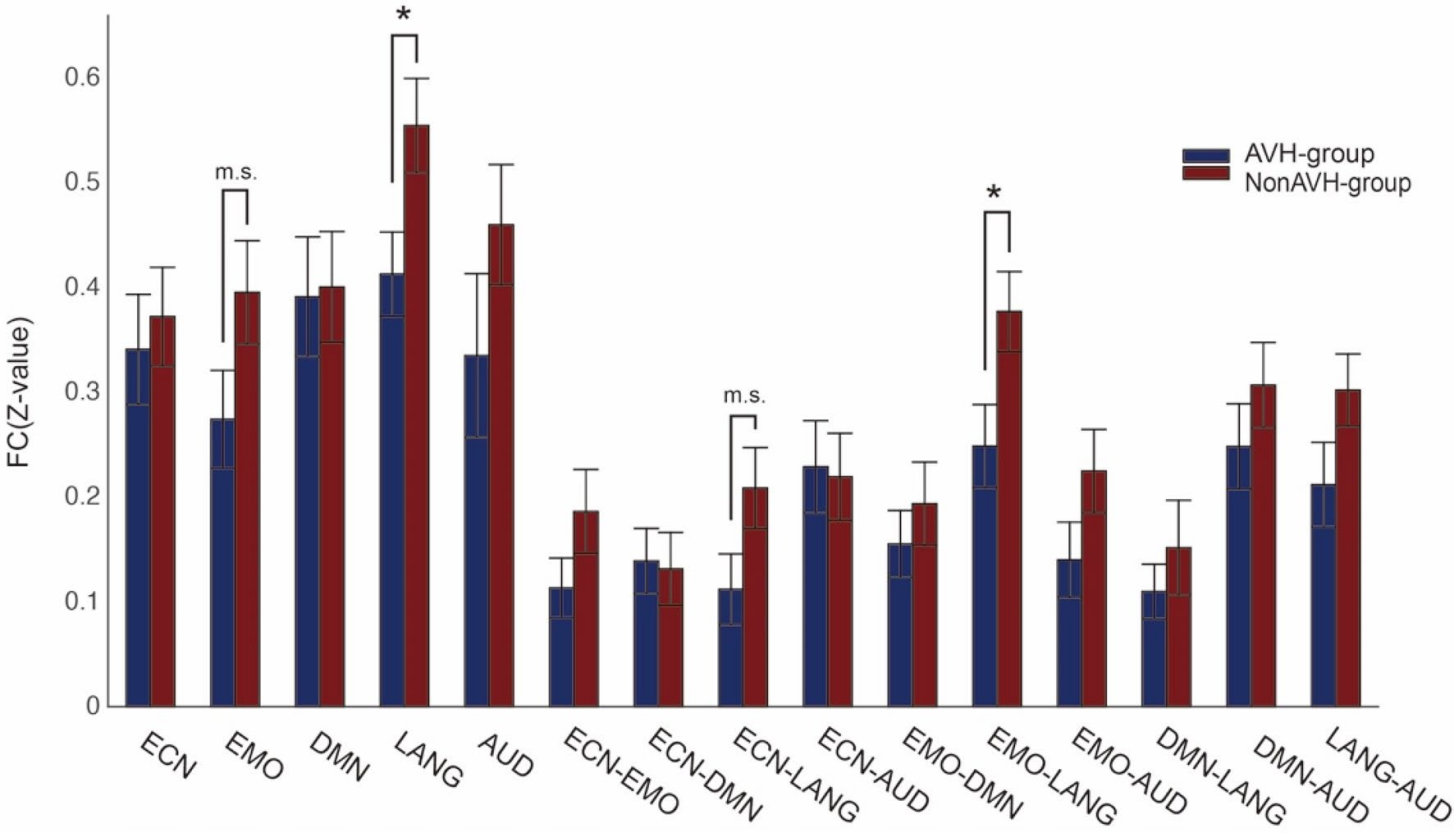
Static inter- and intra-network connectivity between five core brain networks in AVH and non-AVH groups. Notes: *ECN* executive control network, *EMO* emotion network, *DMN* default mode network, *LANG* language network, *AUD* auditory network, *P < 0.05, **P < 0.01, *n.s.* not significant, *m.s* marginally significant (*P* < 0.1)

For static functional connectivity, we examined intra- and inter-network FC. We used intra-network connectivity to investigate the communication between regions within each particular functional network. The two-sample *t* test showed that AVH patients had a decreased connectivity in the language network compared with non-AVH group (*t*(37) =  – 2.348, *p* = 0.024) (Fig. 8). Comparison between the two groups in male patients showed a marginally significant difference (*t*(28) =  – 2.031, *p* = 0.052). Spearman correlation analysis showed no significant correlation between P3 and connectivity in the language network (*r* = 0.031, *p* = 0.932) in the AVH group, which is in line with our data in which P3 is not specific for measuring AVH.

Regarding the inter-network connectivity, AVH patients showed decreased connectivity between the emotion network and the language network (*t*(37) =  – 2.288, *p* = 0.028) (Fig. 8). Repeated analysis in male patients showed a similar but marginally significant difference (*t*(28) =  − 1.873, *p* = 0.071). Again, Spearman correlation analysis showed no significant correlation between P3 and connectivity between the emotion network and the language network (*r* = 0.094, *p* = 0.797) in the AVH group.

### Quality control to validate the main results

Dynamic analysis could be affected by different scanning parameters. To be sure that our findings are not driven by difference in scanning parameters, we compared the dwell time (State 3 and 6), and transition probability (from State 3 to 6 and from State 6 to itself) between different studies using ANCOVA with TR as a factor and AVH as covariate. The results showed that the main effects of TRs were not significant (all *F* (2, 35) < 2.26, all *p* > 0.12), which suggested that different TRs did not significantly affect these measurements in dynamic analysis. To regress out the difference of TRs, we also did the group comparisons of dwell time, translation probability, and static and dynamic connectivity using ANCOVA with AVH as a factor and TRs as covariate. After controlling TRs, the main effects of AVH still remain. For dynamic dwelling and switching, AVH group showed higher dwell time during State 3 (*p* = 0.069), lower dwell time during State 6 (*p* = 0.014), and lower probability to switch from State 3 into State 6 (*p* = 0.076) and from State 6 to itself (*p* = 0.002). For the dynamic connectivity, AVH group decreased connectivity within the auditory network (*p* = 0.025) and between the executive control and language network (*p* = 0.012) during State 3, decreased connectivity within the language network (*p* = 0.024) during State 6. For static connectivity, AVH group showed decreased connectivity in the language network (*p* = 0.009) and between the emotion and language networks (*p* = 0.009).

In the initial analysis, we identified volumes with frame-wise displacement (FD) parameter > 0.5 and we replaced the fMRI values in this volume by a linear interpolation (see methods section). To examine the potential impacts of head motion correction procedure on our findings and rule out possible spurious source driven by head motion, we have performed the following four-step robustness control check.

First, we investigated the difference in the number of outliers per group using permutation test. The group comparisons between AVH and non-AVH group showed no difference of the number of outliers between the two groups (*diff* = 8.181, *p* = 0.407, see Figure S5). Second, initially, we excluded two subjects based on an extent of head motion (see Methods). To further investigate this, in a more stringent control analysis, we further excluded three subjects who had relatively larger number of motion outliers (more than 15% (30 out of 200 volumes) time points (41, 34, 54 out of 200 volumes) considered as outliers (FD > 0.5), which were replaced by linear interpolation), our main findings remained almost the same (see page 39, line 862–872 in the supplement materials) (see Figure S5). Third, in place of interpolation (which we performed in the initial analysis), we now removed/scrubbed the volumes which were considered as motion outliers (FD > 0.5) without filling for them. The main findings remained (see page 40, line 874–882 in the supplement materials). Fourth, we also performed ANCOVA to consider the group (AVH vs non-AVH) as the main factor and the mean of head motion parameter (FD), age, and drug usage as covariates. Again, our main results remained significant after controlling for these covariates (see Table S1). Together, we performed four stringent validation analyses to investigate robustness of our findings. We conclude that the difference between groups in terms of dwell time and switching probability is not driven by the motion correction procedure.

Another issue concerns the choice of window length. In the present study, we used window length as 22 TRs by following the Allen’s study (Allen et al. 2014), which is also consistent with Hutchison’s suggestion that window lengths around 30–60 s can produce robust results in the conventional acquisitions (Hutchison et al. 2013). We also chose window length as 15, 20, and 25 TRs to further check how window length may affect our findings. The results showed that using these different window lengths can replicate our main findings (window length as 22 TRs) including dynamic brain state patterns, dwell times, and functional connectivity in dynamic states (see Figure S3–4).

## Discussion

In this study, we examined dynamic intra- and inter-network functional interactions in relationship to AVH in patients with schizophrenia. Importantly, we also investigated how AVH patients dwelled in and switched between specific brain states (i.e., the ‘network-antagonistic’ and segmented brain states) and other brain states. Given the dynamic nature of hallucinations, dynamic connectivity analysis may be of special interest to elucidate the neural basis of hallucinations. The results showed that AVH patients spent less time in a ‘network-antagonistic’ brain state which showed anti-correlation between the DMN and the language network, and had a lower probability to switch into this state. This may imply that language processing is less distinct from resting-state processes (e.g., the DMN represents) in these patients, which could explain higher levels of verbal intrusions in people with hallucinatory predisposition (Waters et al. 2012). In addition, AVH patients had decreased connectivity within the language network during the ‘network-antagonistic’ brain state, weaker connectivity in the auditory network, as well as between the executive control network and the language network in the segregated brain state. Thus, our results suggest altered interaction among brain networks during certain brain states in AVH, which may be of relevance to fluctuations of hallucinatory activity. These findings validate and extend previous models of hallucinations (Allen et al. 2008a, b; Northoff and Qin 2011; Waters et al. 2012) by corroborating the relevance of the language and the executive control network. Beyond that, we provide novel evidence of altered brain dynamics in AVH, including dwelling and switching between specific brain states.

### Less dwelling in a ‘network antagonistic' brain state in AVH

We examined the dynamic of brain networks during resting-state in AVH and non-AVH patients and pinpointed two important brain states: a segregated brain state and a ‘network-antagonistic’ brain state. We found the lower cluster coefficient and longer shortest path length in State 3, which indicated that the brain was disconnected. In particular, lower clustering coefficient indicated fewer connections among the nearest neighbors of a node. Longer shortest path length indicated that more steps are needed to transmit information from one node to another. In line with the disconnection hypothesis of schizophrenia (Stephan et al. 2009), many previous resting-state fMRI findings have suggested that the segregated brain state, similar with the State 3, was more specific in patients with schizophrenia compared to healthy controls. During the ‘network-antagonistic’ brain state (State 6), DMN showed anti-correlation with the language network. The ‘network-antagonistic’ connectivity pattern in State 6 is very similar with an anti-correlated activation pattern (i.e., antagonism) between the DMN (e.g., vmPFC and PCC) and task-positive networks (e.g., dlPFC and IPS) in healthy people (Fox et al. 2005).

The group comparisons showed that AVH group dwelled less time in State 6 and had a lower probability of switching from State 6 to itself. The dominant feature of State 6, antagonism between the DMN and the language network, may play an important role in functional specialization of these networks and thereby minimizing overlap that could engender confusion as to the source and nature of information processed. Less dwelling in State 6 may suggest that AVH patients are not able to stay in a ‘healthy’ brain state which can distinguish self-related processing (putatively related to DMN) and verbal processing (putatively related to the language network). Our findings is line with extensive evidence which have suggested that schizophrenia patients showed altered antagonism between the DMN and other networks, e.g., switching from negative to positive correlation (Anticevic et al. 2012). In the context of AVH, the DMN was considered to be involved in self-related processing and memory replay of language information, which was proposed to be directly linked to production of AVH (Northoff and Qin 2011; Northoff 2014; Ćurčić-Blake et al. 2017a). The alteration of antagonism between the DMN and other networks has been proposed to contribute to wrong assignment of saliency to internal speech in hallucinations (Palaniyappan and Liddle 2012). Similarly, the ‘resting-state hypotheses’ of AVH (Northoff and Qin 2011) proposed that less suppression of the DMN and increased connectivity of the DMN play important roles in spontaneous over-perception in auditory regions, which has been supported by several experimental studies (Whitfield-Gabrieli et al. 2009; Broyd et al. 2009). Extending these findings of altered antagonism between the DMN and other networks in static functional connectivity studies, our study is the first to provide novel evidence that patients with AVH showed altered dynamic nature of antagonism between the DMN and the language network, which might contribute to transient experience of AVH.

Additionally, the anti-correlation between the DMN and the language network might be related to predictive coding. In the predictive coding theory, Carhart-Harris and Friston (Carhart-Harris and Friston, 2010) proposed that the brain is hierarchical, with the DMN at the top and the ECN at intermediate levels, above sensory cortices. Recurrent information–transmission and reciprocity excitation/inhibition between these systems result in self-organized stable (balanced) activation patterns to enable efficient prediction, perception and error-based learning. Therefore, it is reasonable to speculate that the reciprocity excitation/inhibition between the DMN and the language network may be involved in balancing activation patterns to enable efficient communication between prediction (DMN) and auditory verbal perception. However, our findings are insufficient to either directly support or refute the predictive coding theory. This putative linkage remains to be studied in more details, for instance, by combining a semantic prediction task with the hierarchical Bayesian model and neuroimaging approaches (Vercammen and Aleman 2010; Powers et al. 2017).

### Decreased interaction within the auditory network and between the language and executive control networks in certain dynamic states in AVH

During the segregated brain state (State 3), which showed lower cluster coefficient and longer shortest path length, we found that AVH patients showed much lower connectivity in the auditory network compared with non-AVH patients. The STG in the auditory network is a core region involved in AVH, which serves as a hub in the AVH-related brain system. Hypo-activation in this region is important for bottom-up ‘over-perceptualiztion’ in the development of AVH (Allen et al. 2008a, b). This speculative hypo-activation of STG may contribute to disrupted connectivity between STG and MTG in the auditory network during this segmented state.

Interestingly, dysconnectivity of the auditory network was not present in the static resting state (see below) but in particular during the segregated brain state (State 3), which may mediate the actual occurrence of hallucinations (cf. Jardri et al. 2011). This hypothesis needs to be examined in a future study which tracks hallucination state "on-line" (i.e., during brain scanning). Interestingly, these different findings from static and dynamic connectivity analysis suggest that dynamic connectivity approach can unveil abnormality of brain networks which static connectivity approaches cannot provide (Hutchison et al. 2013; Damaraju et al. 2014), which may provide an important possible hypothesis of that this state may correspond to the state hallucinations. In this segregated state, we also found decreased connectivity between the executive control network and the language network, which may be related to dysfunction of top–down control of language processing in AVH. These results support models implying altered fronto-parietal top-down control in hallucinations (Allen et al. 2008a, b; Hugdahl 2009).

During the ‘network-antagonistic’ brain state (State 6), reduced interaction in the language network occurred, where the DMN showed less anti-correlation with the language network in patients with AVH. This may indicate that less antagonism between the DMN and the language network may be related to dysfunction of either network, or both being less distinctive in terms of specialized function, by which the DMN could be "invaded" by intrusions of language information from memory (Ćurčić-Blake et al. 2017; Northoff 2014; Northoff and Qin 2011).

### Static functional connectivity and AVH

Static functional connectivity analysis showed that AVH patients had decreased connectivity within the language network and between the language and emotion networks. Our results are in line with altered connectivity between key language regions and emotional regions, which may be involved in disrupted communication of language information (Lawrie et al. 2002; Vercammen et al. 2010) and emotion-related attention (Aleman and Kahn 2005; Smith et al. 2006; Allen et al. 2008a; Kang et al. 2009; Escartí et al. 2010) in AVH. Although findings of our static connectivity are highly consistent with the previous literature, we should remain cautious about drawing firm conclusion, because they did not survive after multiple comparison correction.

### Disconnection hypothesis of schizophrenia and hallucinations

Our dynamic and static connectivity analyses have showed that AVH in schizophrenia was characterized by weakened connectivity distributed among core AVH-related brain networks including the auditory, the language and the executive control, and the emotion networks. In addition, AVH patients tend to spend more time in a segregated brain state (State 3). In line with the disconnection hypothesis of schizophrenia (Stephan et al. 2009), many previous resting-state fMRI studies found decreased functional connectivity in patients with schizophrenia compared to healthy controls (Liang et al. 2006; Lynall et al. 2010; Pettersson-Yeo et al. 2011). More specifically, patients with auditory verbal hallucination showed disrupted functional connectivity among frontal, parietal, and temporal lobes (Ćurčić-Blake et al. 2017b). Importantly, corollary discharge theory has suggested that disconnection in patients with hallucinations might underline reduced communication between top-down systems (i.e., the executive control networks) and the sensory-specific systems including the language and auditory networks (Fletcher and Frith 2009). The disrupted connection might lead to a failure in the prediction-driven attenuation of sensory consequence of internal speech and difficulties in distinguishing internally from externally generated stimuli. This is in line with our finding of reduced connectivity among multiple brain networks. However, noteworthy, this hypothesis of association between reduced connectivity among brain networks and disrupted corollary discharge should be explicitly examined in studies in which cognitive tasks are carefully designed to target cognitive processes directly engaging interaction between prediction regions and perception/sensory regions.

It might be valuable to discuss our finding in the context of Lefebvre et al.’s study. They used the DCM to examine the causal interaction between brain networks during different phases of hallucination experiences. They found that memory-based sensory input from the hippocampus to the salience network increases during the “on” period of hallucinations and a takeover of the CEN in favor of a voluntary process is associated with the “end” period of hallucinations. In our study, we found that patients with hallucinations spent less time in a State 6 characterized by the anti-correlation (i.e., antagonism) between the DMN and the language network. The antagonism state may serve as a healthy brain state which protects patients from confusion between self-related processing (putatively related to the DMN) and language processing (putatively related to the language network). This state may be related to the “end” state in Lefebvre study, which is characterized by a takeover of the ECN. In State 6, we also found the reduced connectivity between the DMN and the ECN (compared to other states). It is possible that interaction between the ECN and the DMN contributes to the proper antagonism between the DMN and the language network, which may protect people from hallucinating.

## Limitations

Several potential limitations of our study should be noted. First, due to difficulty of collecting clinical patients, especially patients without hallucinations, the sample size in the present study is moderate, which limits statistical power. However, the sample is still relatively large comparing with the other AVH studies (cf. Ćurčić-Blake et al. 2017). Second, we used data from 3 datasets with different scanning parameters. The major difference is in TR which varies between 1.5 and 2.3 s. Nevertheless, we checked if this difference affected the dwelling time and transition possibility, actually, this was not the case (see the section Quality control). To be on the conservative side, we compared these neural measurements between the two groups while correcting for different TRs, and the results remained the same (see details in the control analysis). Third, although the limited number of females in the AVH and non-AVH groups did not allow us to directly examine effect of gender on the results, we performed group comparisons for males with and without AVH and found that the main results still held up. It would be instructive to examine whether our findings can be generalized to female patients in future investigations. Fourth, for the sliding window approach, selecting parameters of windows length and step is still under debate, but selection of these parameters in the present study is supported by the previous experiential and stimulation studies (Damaraju et al. 2014; Allen et al. 2014). Finally, given that we focused on the trait rather than state of hallucinations, we did not find a significant correlation between P3 and dynamic brain measurements. In the future, samples with active hallucinations in the preceding days (as compared to patients without active hallucinations) should be compared, including hallucination measures that are more comprehensive regarding AVH. Examples of such measures are the PSYRATS and the AVHRS (Haddock et al. 1999; Steenhuis et al. 2019).

## Conclusion

We found that patients with a recent history of AVH showed altered static and dynamic connectivity patterns within and between core brain networks compared to patients without a recent history of AVH during resting state. AVH patients showed dysconnectivity in the language network and the auditory network, between the emotion and language networks, and between the executive control and the language networks. The disconnection of these brain networks during static and dynamic connectivity validates and extends previous brain models of hallucinations which suggested altered interaction between the language perception networks and control networks. More importantly, AVH patients dynamically dwelled less time in the brain state where the DMN has strong antagonism with the language network, which may be crucial for avoiding memory replay of language information. This information of alterations in dynamics of brain networks in AVH may potentially inform cognitive models that aid the development of new coping strategies for patients to control AVH.

## Electronic supplementary material

Below is the link to the electronic supplementary material.

Supplementary file1 (DOCX 936 kb)

## Data Availability

Codes used in this study are available from the corresponding author on request.
