# Supplementary material for: Abnormal dynamic resting-state brain network organization in auditory verbal hallucination
Source: Brain Struct Funct. 2020 Aug 19;225(8):2315–30. doi: 10.1007/s00429-020-02119-1 (PMC7544708; doi:10.1007/s00429-020-02119-1)
Supplement: Supplementary file 1 — Supplementary file1 (DOCX 936 kb) [file 429_2020_2119_MOESM1_ESM.docx]

**Supplemental Texts**

**Determining cluster number in k-means**

We used Dunn index ( ${DI}_{m}= \min_{0\leq i<j\leq m} \delta(C_{i},c_{j})/\max_{1\leq k\leq m} \Delta_{k}$) to evaluate clustering performance and determinate number of clusters, the m is cluster number which the DI is the highest, $\delta$ is distance between cluster i and cluster j, $\Delta$ is distance within cluster k. The approach aimed to identify a number of clusters with minimum variances between members within the cluster and maximum variances between clusters. By using Dunn index, we firstly selected number of clusters as 4 and 6 across all the patients because they have comparably maximal Dunn values (see Figure S1). After that, we examined the FC patterns in 4 states and 6 states. Eventually we chose 6 states, because it included a state with a meaningful connectivity pattern (i.e. anti-correlation between the DMN and task positive networks, so called antagonistic state, Figure S2), consistent with previous connectivity studies (Fox et al., 2005).

**Examining impacts of head motion on the main results**

To stringently examine whether our results are affected by scrubbing approach (i.e. the linear interpolation), we performed three kinds of validation analyses to test robustness of our findings. In the first analysis, we excluded the three participants with relatively but acceptable higher head motions (more than 15% time points (add the number here) considered as outliers, which were replaced by linear interpolation) and re-do the clustering analysis and group comparisons of dwell time, transition probability and functional connectivity. After excluding these three participants, the main effects of AVH in original analysis still remained. For dynamic dwelling and switching, AVH group showed significantly lower dwell time during State 6 (*p* = 0.010), lower probability to switch from State 6 to itself (*p* = 0.004). For the dynamic connectivity, AVH group decreased connectivity within the auditory network (*p* = 0.011) during State 3, a trends of decreased connectivity within the language network (*p* = 0.059) during State 6. For static connectivity, AVH group showed significantly decreased connectivity in the language network (*p* = 0.0453) and between the emotion and language networks (*p* = 0.0245).

In the second control analysis, we examined the impacts of head motion by removing all the ‘outlier’ volumes to re-do all the analysis. The main results shown in the original analysis still hold the same. For dynamic dwelling and switching, AVH group showed lower dwell time during State 6 (*p* = 0.009), lower probability to switch from State 6 to itself (*p* = 0.0029). For the dynamic connectivity, AVH group showed a trend of decreased connectivity within the auditory network (*p* = 0.052) and a significant reduced connectivity between the executive control network and the language network (*p* = 0.0136) during State 3.

In the third analysis, according to the suggestions from the second reviewer, we performed ACOVA to consider the AVH group as the main factor and head motion parameter (FD), age and drug usage as covariates, our main results still hold same after controlling the these covariates (see **Table S1**).

Together, by performing four very stringent validation analyses, the results after very careful consideration of head motions remain almost the same with main results shown in our main manuscripts. It excludes the possibility that interpolation analyses and head motion have significant impacts on the main results.

**Supplemental Figures**


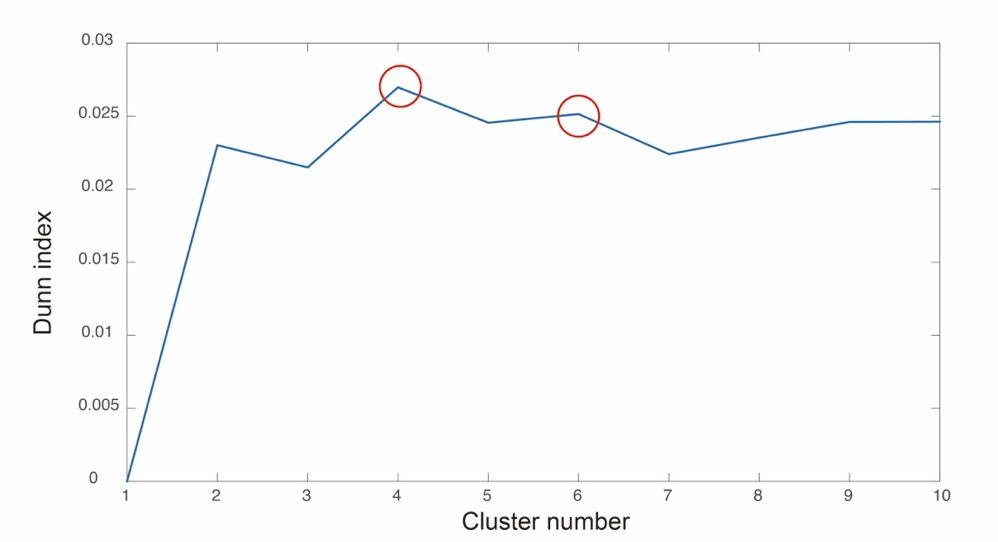


**Figure S1. Dunn index changing with cluster number**


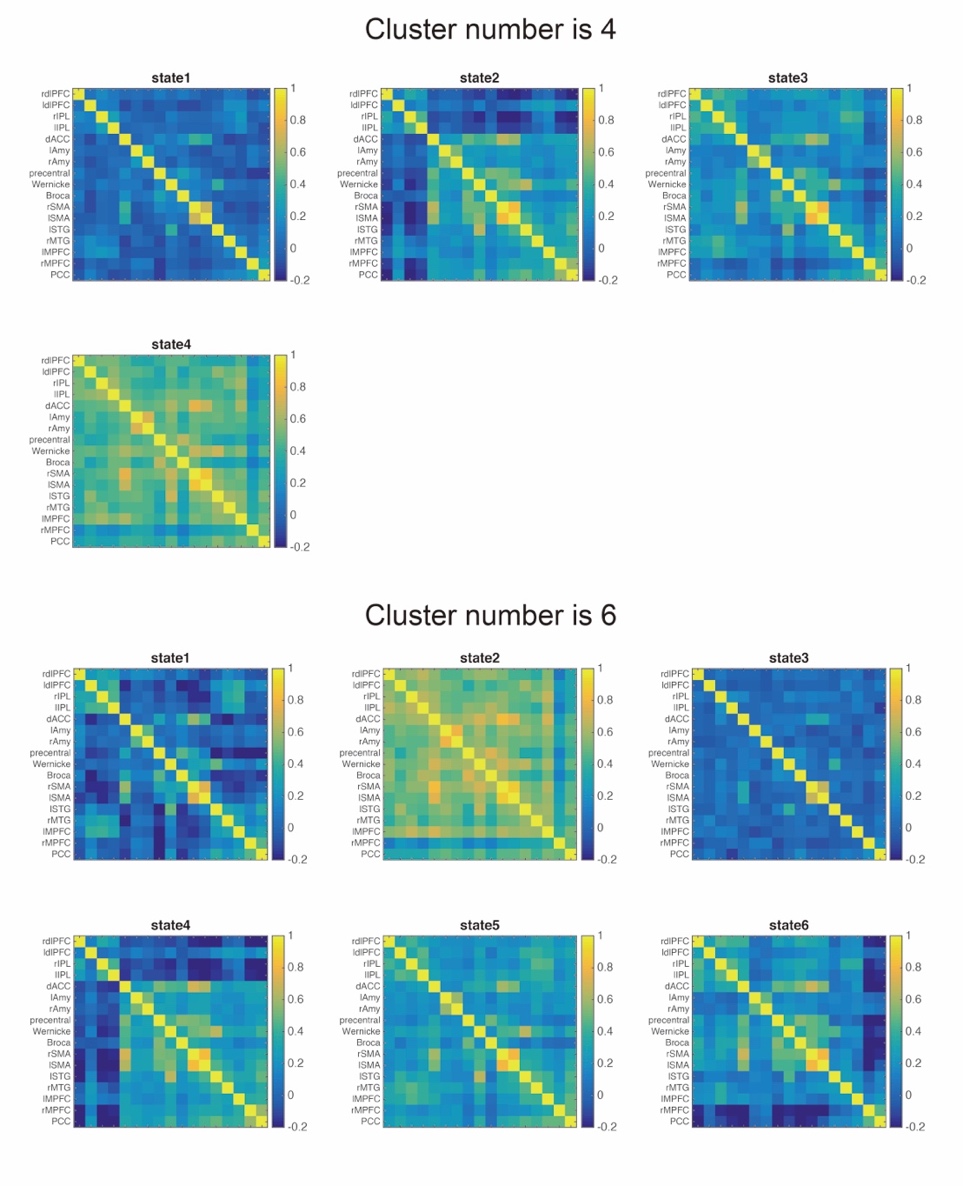


**Figure S2. Connectivity patterns during four and six brain states.**

~
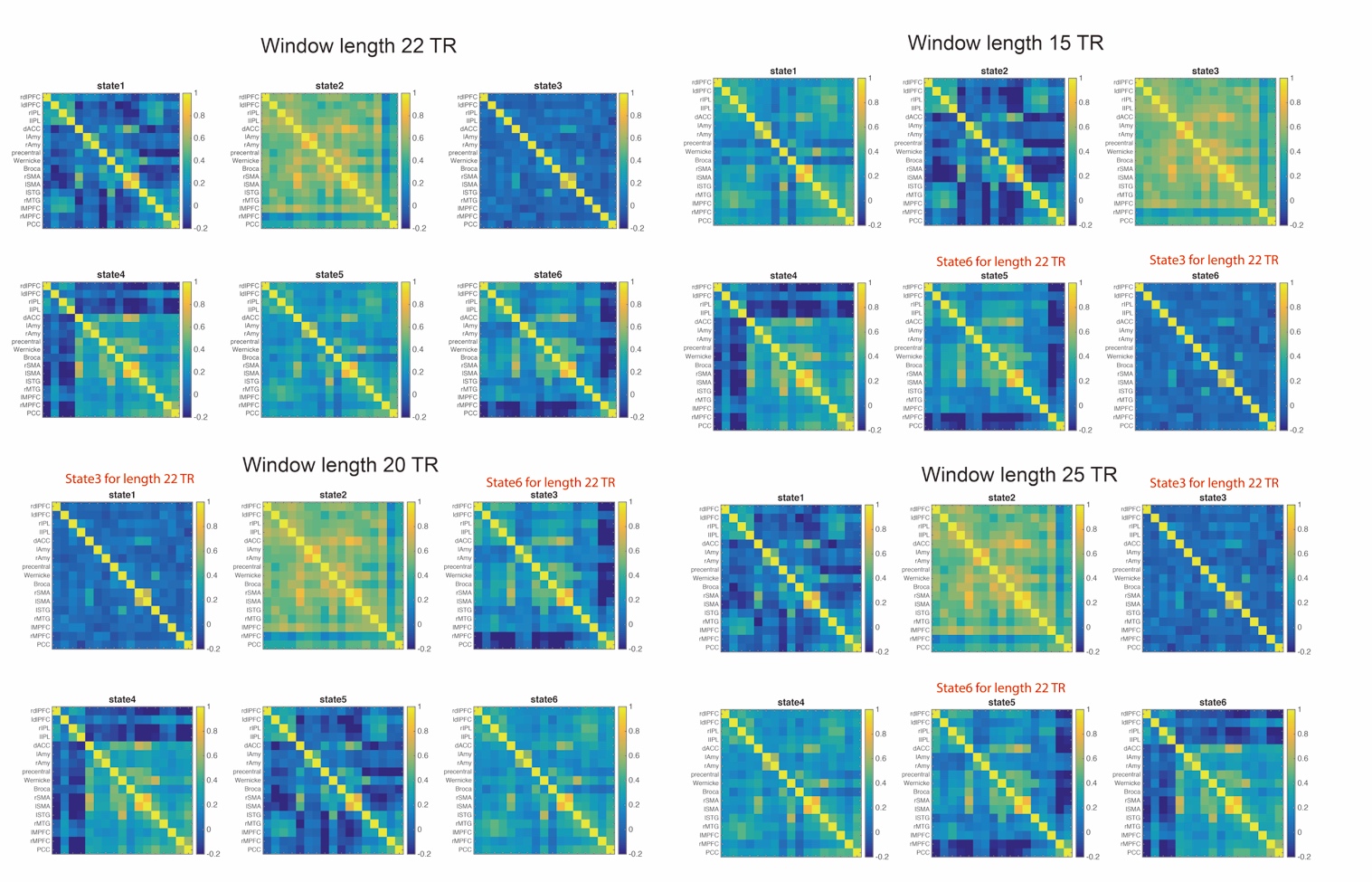


**Figure S3 Connectivity patterns of six brain states across different window lengths.**


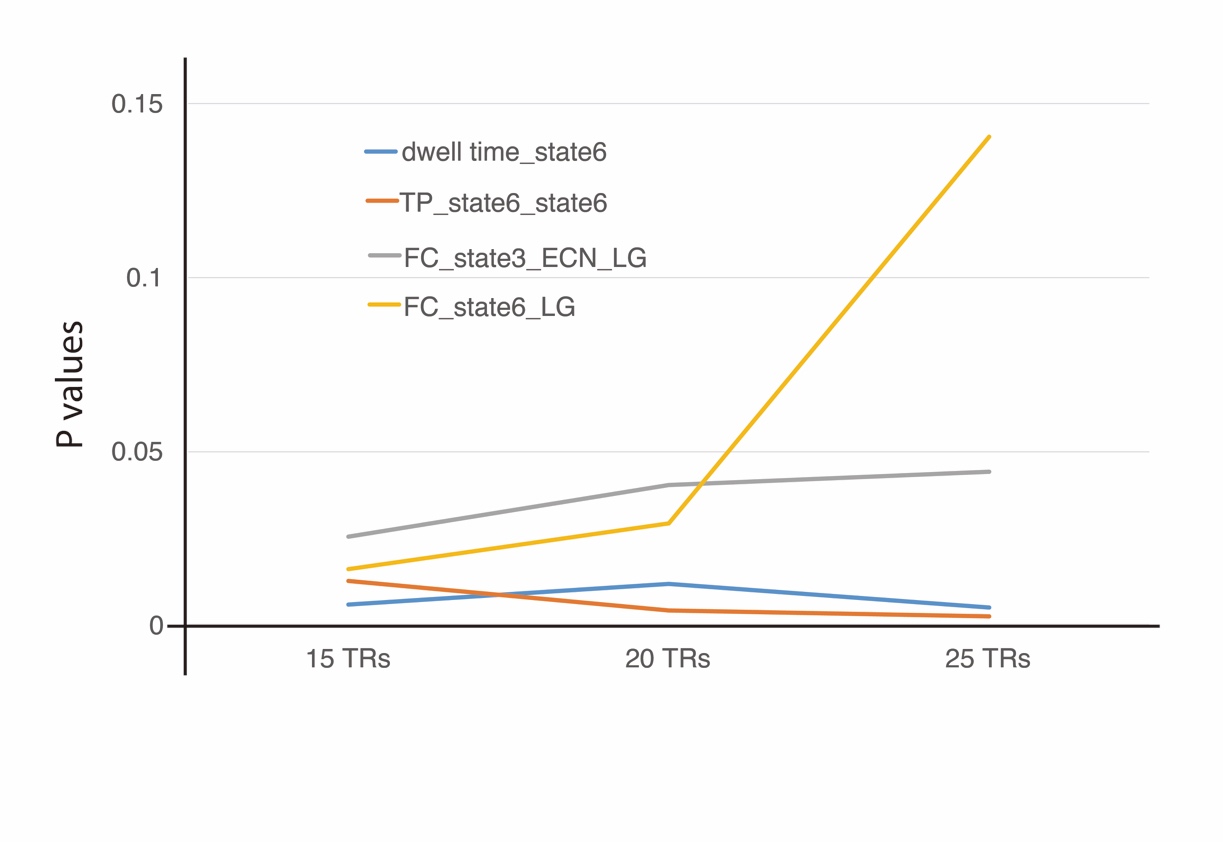


**Figure S4 P values of group comparisons for dwell time, transition probability and function connectivity in dynamic states across different window lengths. Notes: FC, functional connectivity; ECN, executive control network; LG, language network.**


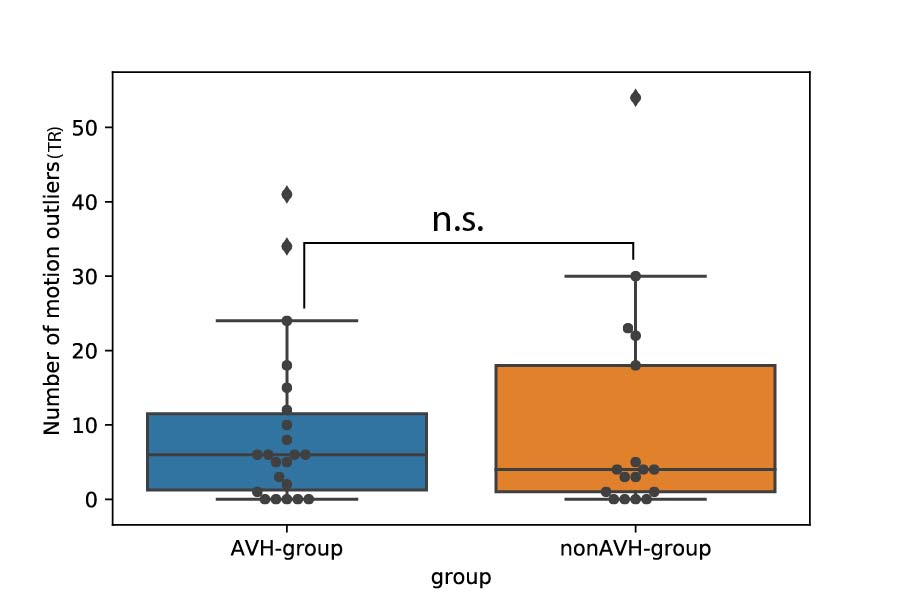


**Figure S5. Comparison of head motion outliers between the AVH group and nonAVH group**

**Table S1 The main effect of AVH group and effects of age, drug dose and head motion on neural measurements.**

|  | AVH group (F value/p value) | Age (F value/p value) | Drug dose (F value/p value) | Head motion (F value/p value) |
| --- | --- | --- | --- | --- |
| dwell_time_s6 | 7.145/0.011 | 0.066/0.799 | 0.178/0.676 | 0.997/0.325 |
| TP_3_6 | 3.568/0.067 | 0.062/0.805 | 0.319/0.576 | 0.695/0.410 |
| TP_6_6 | 9.991/0.003 | 0.630/0.433 | 0.850/0.363 | 0.008/0.929 |
| AUD_FC_s3 | 4.385/0.048 | 0.012/0.914 | 0.0006/0.981 | 0.001/0.974 |
| ECN_LG_s3 | 7.539/0.012 | 2.106/0.161 | 0.193/0.665 | 4.674/0.042 |
| LG_FC_s6 | 3.935/0.058 | 0.027/0.870 | 0.323/0.575 | 0.508/0.482 |
| LG_FC | 4.747/0.036 | 1.095/0.303 | 0.039/0.845 | 0.780/0.383 |
| EMO_LG_FC | 5.279/0.028 | 4.744/0.036 | 0.066/0.798 | 1.504/0.229 |

**Notes: TP, transition probability; AUD, auditory network; FC, functional connectivity; ECN, executive control network; LG, language network; EMO, emotional network.**
